# Next-Generation Predictive Microbiology: A Software Platform Combining Two-Step, One-Step and Machine Learning Modelling

**DOI:** 10.3390/foods14183158

**Published:** 2025-09-10

**Authors:** Fatih Tarlak, Büşra Betül Şimşek, Melissa Şahin, Fernando Pérez-Rodríguez

**Affiliations:** 1 Department of Bioengineering, Gebze Technical University, 41400 Gebze, Turkey; b.simsek2025@gtu.edu.tr (B.B.Ş.); msahin2019@gtu.edu.tr (M.Ş.); 2 Department of Food Science and Technology, UIC Zoonosis y Enfermedades Emergentes ENZOEM, International Campus of Excellence in the AgriFood Sector (CeiA3), University of Córdoba, 14014 Córdoba, Spain; b42perof@uco.es

**Keywords:** predictive microbiology, machine learning, microbial growth, growth inhibition, software platform, data-driven modelling

## Abstract

Microbial growth and inhibition are complex biological processes influenced by diverse environmental and chemical factors, posing challenges for accurate modelling and prediction. Traditional mechanistic models often struggle to capture the nonlinear and multidimensional interactions inherent in real-world food systems, especially when multiple environmental variables and inhibitors are involved. This study presents the development of a novel, dynamic software platform that integrates classical predictive microbiology models—including both one-step and two-step frameworks—with advanced machine learning (ML) methods such as Support Vector Regression, Random Forest Regression, and Gaussian Process Regression. Uniquely, this platform enables direct comparisons between two-step and one-step modelling approaches across four widely used growth models (modified Gompertz, Logistic, Baranyi, and Huang) and three inhibition models (Log-Linear, Log-Linear + Tail, and Weibull), offering unprecedented flexibility for model evaluation and selection. Furthermore, the platform incorporates ML-based modelling for both microbial growth and inhibition, expanding predictive capabilities beyond traditional parametric frameworks. Validation against experimental and literature datasets demonstrated the platform’s high predictive accuracy and robustness, with machine learning models, particularly Gaussian Process Regression and Random Forest Regression, outperforming classical models. This versatile platform provides a powerful, data-driven decision-support tool for researchers, industry professionals, and regulatory bodies in areas such as food safety management, shelf-life estimation, antimicrobial testing, and environmental monitoring. Future work will focus on further optimization, integration with large public microbial databases, and expanding applications in emerging fields of predictive microbiology.

## 1. Introduction

Predictive microbiology integrates microbiological knowledge with mathematical and computational tools to quantify and forecast microbial growth, survival and inactivation in foods and other matrices, supporting food-safety management, shelf-life setting and risk assessment [1,2].

Two biological processes dominate this field: growth, the sigmoidal increase in cell numbers under favorable conditions, and inhibition (or inactivation), the decline in viable cells under stresses such as low temperature, low pH or disinfectants [3,4].

Conventionally, predictive microbiology relies on a two-layer framework. Primary models—such as the modified Gompertz, Logistic, Baranyi or Huang functions for growth dynamics, and Log-linear, Log-linear with tail, or Weibull equations for inactivation—are used to describe microbial population changes as a function of time under constant conditions [5,6,7,8]. Secondary models, including the Ratkowsky square-root model and the Cardinal Parameter Model, then relate the kinetic parameters obtained from primary fits (e.g., µ_max_, lag time, or δ) to environmental variables such as temperature, pH, or water activity [9,10]. In practice, this produces a two-step workflow (fit primary model → regress parameters against environment), but this strategy carries a major weakness: estimation errors from the primary stage are propagated to the secondary stage, which amplifies overall uncertainty, especially with sparse or noisy data and in systems where environmental effects are strongly nonlinear and interactive [11].

To address these drawbacks, one-step (global) modelling has been proposed, where environmental terms are embedded directly into the primary equations and all parameters are estimated simultaneously [3,12,13]. In principle, this approach can yield more stable and biologically interpretable fits. However, it also introduces new challenges: multi-parameter optimization is computationally demanding, prone to convergence at local minima, and highly sensitive to perturbations in the data.

These difficulties are not unique to global fitting but mirror the well-known limitations of classical models themselves. Primary growth models such as the modified Gompertz, Logistic, Baranyi, and Huang functions [5,6,7], as well as inactivation models including the Log-linear, Weibull, and Log-linear with Tail formulations [8,14], are all tied to fixed functional forms. While such equations can capture general patterns, they often require ad hoc modifications to describe deviations such as shoulders, tails, or nonlinear transitions. As a result, whether applied through the conventional two-step workflow or the more integrative one-step approach, both strategies remain constrained by rigid mathematical structures, restricting their ability to represent the nonlinear, multidimensional interactions that characterize microbial behaviour in real food matrices [4,9,11].

Within this context, machine learning models offer a compelling alternative. Regressors such as Support Vector Regression, Random Forests, and Gaussian Processes provide flexibility in capturing nonlinearities and higher-order interactions while simultaneously handling multiple explanatory variables [15,16]. Unlike classical mechanistic approaches that require distinct model formulations for growth and inactivation, machine learning can unify both behaviours within a single predictive framework, thereby reducing workflow complexity and avoiding the rigid assumptions of classical models. Validation against benchmark datasets for microbial growth and inactivation has demonstrated that such methods can reproduce, and sometimes exceed, the predictive performance of traditional models [17,18,19].

Collectively, the shortcomings of the classical two-step and one-step approaches—error propagation, instability, and restricted generalizability—establish a strong rationale for adopting machine learning as a more flexible, accurate, and forward-looking paradigm for predictive microbiology.

This study presents a dynamic software platform that integrates classical predictive microbiology models with modern machine learning (ML) approaches in a single, interactive environment. The platform enables side-by-side comparisons between one-step and two-step modelling workflows across four commonly used primary growth models (modified Gompertz, Logistic, Baranyi, and Huang) and three inactivation models (Log-Linear, Log-Linear + Tail, and Weibull). In addition, it incorporates ML algorithms such as Support Vector Regression, Random Forest Regression, and Gaussian Process Regression for both growth and inactivation prediction. By combining mechanistic and data-driven approaches, the platform seeks to provide both interpretable insights—useful for regulatory compliance and shelf-life estimation—and the ability to handle complex, multivariable datasets, which may support real-time applications in food safety. Benchmark datasets for microbial growth [17,18] and chlorine-based surface inactivation [19] were used to demonstrate the functionality of the platform, showing that it can reproduce established modelling approaches while offering added features such as uncertainty visualization, interactive diagnostics, and report generation. Through its unified API (Application Programming Interface) and user interface, the tool is designed to facilitate scenario testing, model comparison, and decision support for researchers, industry professionals, and regulatory stakeholders.

## 2. Material and Methods

A dynamic software platform (version 1.0) was developed to model microbial population dynamics by combining classical mechanistic equations with modern machine learning regression techniques. The platform was implemented in Python 3.12, using NumPy and SciPy 1.13 for numerical computations, scikit-learn 1.6 for ML algorithms, and Flask 3.0 to create a web-based interface. Users can submit raw growth or inactivation data to dedicated endpoints, and the platform returns fitted parameters, uncertainty estimates, and goodness-of-fit statistics. Interactive plots and downloadable reports are also provided through the web interface. The application is accessible at: https://fatihtarlak.pythonanywhere.com (accessed on 3 September 2025), with stable documentation, sample datasets, and a step-by-step guide available at: https://github.com/ftarlak/FOODS_2025 (accessed on 3 September 2025).

**Figure 1:**
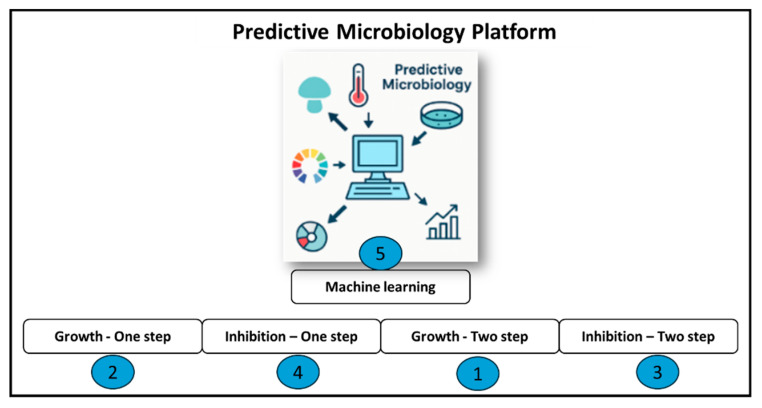
Software for the developed modelling platform.

Figure 1 shows the interface of the Predictive Microbiology Platform features five main buttons, each corresponding to a specific modelling function. The “Growth–Two step” button (1) allows users to fit microbial growth data using a two-step approach, while the “Growth–One step” button (2) performs a similar function with a single-step method. The “Inhibition–Two step” button (3) and “Inhibition–One step” button (4) are designed for modelling microbial inactivation processes using either a two-step or one-step workflow, respectively. At the centre, the “Machine learning” button (5) enables users to apply modern ML regression techniques for predictive modelling, providing a flexible alternative to classical mechanistic equations for growth and inhibition processes. These buttons give users direct access to each modelling endpoint of the platform, streamlining the workflow for data analysis and parameter estimation.

### 2.1. Classical (Traditional) Modelling

In classical predictive microbiology, mechanistic models are used to describe microbial growth and inactivation under specific environmental conditions [1,4]. Growth models such as modified Gompertz, Logistic, Baranyi, and Huang equations capture sigmoidal increases in microbial counts, characterized by parameters like maximum specific growth rate (*μ*) and lag phase (*λ*), often linked to environmental factors through secondary models like the Ratkowsky equation [5,6,7].

In contrast, inactivation models—including Weibull, Log-Linear, and Log-Linear + Tail—describe microbial declines due to stressors such as heat or preservatives, using parameters like the shape factor (p) and D-value (δ) [8,14]. Traditionally, a two-step modelling approach fits primary models first and then relates their parameters to environmental variables through secondary models, but this can lead to error propagation [11]. Alternatively, a one-step approach estimates kinetic and environmental parameters simultaneously, improving stability and biological interpretability, though it requires more complex numerical methods [13].

#### 2.1.1. Growth Models

The modified Gompertz, Logistic, Baranyi and Huang models are among the most commonly used sigmoid functions for modelling bacterial growth behaviour. Under constant environmental conditions, the modified Gompertz and Logistic models are defined by Equation (1) and Equation (2), respectively [5]:

(1) $${x\left(t\right)=x_{0}+(x}_{max}-x_{0})\cdot exp\left\{-exp\left[\frac{r_{max}\cdot e}{{(x}_{max\;}-x_{\;0})}\cdot \left(\lambda -t\right)+1\right]\right\}$$

(2) $$x\left(t\right)=x_{0}+\frac{{(x}_{max}-x_{0})}{\left\{1+\exp\left[\frac{{4\cdot r}_{max}}{{(x}_{max\;}-x_{\;0})}\cdot \left(\lambda -t\right)+2\right]\right\}}$$

where t is the time (h), *x*(*t*) is the bacterial population concentration (log CFU/g) at time t, *x*_0_ is the initial bacterial population concentration (log CFU/g), *x_max_* is the maximum bacterial population concentration (log CFU/g), *r_max_* is the maximum bacterial growth rate (log CFU/h), and *λ* is the lag phase duration (h).

Other widely used primary functions include the Baranyi and Huang models, which are represented by Equation (3) and Equation (4), respectively [6,7]:

(3) $$y\left(t\right)=y_{0}+\mu_{max}F\left(t\right)-ln\left(1+\frac{e^{\mu_{max}F\left(t\right)}-1}{e^{\left(y_{max}-y_{0}\right)}}\right)$$

(4) $$y\left(t\right)=y_{0}+y_{max}-ln(e^{y_{0}}+\left[e^{y_{max}}-e^{y_{0}}\right]\cdot e^{{-\mu }_{max}B\left(t\right)})$$

where *t* is the time (h), *y*(*t*) is the bacterial population concentration (ln CFU/g) at time *t*, *y*_0_ is the initial bacterial concentration (ln CFU/g), *yₘₐₓ* is the maximum bacterial population concentration (ln CFU/g), *µₘₐₓ* is the maximum specific bacterial growth rate (1/h), and λ is the lag phase duration (h). *F*(*t*) and *B*(*t*) are adjustment functions as described by Baranyi and Roberts [6] and Huang [7]. The modified Gompertz and Logistic models employ the common logarithm (log_10_), whereas the Baranyi and Huang models use the natural logarithm (log_e_, ln). Accordingly, the growth rate values (*r_max_*) estimated from the modified Gompertz and Logistic models were converted to *µ_max_* by multiplying by ln(10) [20].

Secondary models, applied after primary curves are fitted, quantify how water activity, acidity and temperature modify growth-model parameters [20,21]. This linkage is vital for food-safety and quality management [22]. A widely used example is the Ratkowsky square-root equation, which relates temperature directly to the maximum specific growth rate [9]:

(5) $${\mu_{max}=\;b_{1}\left(Independent\;variable-{Independent\;variable}_{min}\right)}^{2}$$

where *µ_max_* is the maximum specific growth rate (1/h) obtained from the primary model; *Independent variable* denotes an environmental factor (water activity, pH, or temperature), and *Independent variable_min_* is the minimum value of that factor below which growth ceases; *b*_1_ is the regression coefficient.

Additionally, *λ* (lag phase duration) was defined as a function of *µ_max_* with respect to temperature using Equation (6) [23]:

(6) $$\lambda \;=\frac{b_{2}}{\mu_{max}\left(Independent\;variable\right)\;}$$

where *b*_2_ is the regression coefficient, *µ_max_* (Independent variable) is a function of temperature, water activity or acidity, which leads *λ* to be defined as a function of storage temperature, water activity or acidity.

#### 2.1.2. Inhibition Models

In predictive microbiology, inactivation models describe how microbial populations decline when exposed to adverse conditions such as heat, chemical preservatives, or other stressors. Whereas growth models follow rising cell counts, inactivation models capture the time-dependent reduction in viable cells and include parameters that reflect the kinetics and shape of the resulting death curves. Among the most common formulations is the Log-Linear model, which assumes a constant inactivation rate; its mathematical form is given in Equation (7):

(7) $$\log(\;N)\;=\;\log\left(N_{0}\right)\;-k*t$$

where N is the microbial population at time *t*, N_0_ is the initial population, and *k* is the inactivation rate constant.

To account for situations where a fraction of the microbial population persists despite prolonged treatment, the Log-Linear + Tail model is used. This model describes a survival tail and is expressed as Equation (8):

(8) $$log\left(N\right)=\left\{\log\left(N_{0}\right)-\log\left(N_{res}\right)\right\}*exp\left(-k*t\right)+\log\left(N_{res}\right)$$

where N*_res_* is the residual population density that survives the treatment.

Additionally, the Weibull model provides a flexible framework capable of representing various curve shapes, including concave or convex survival curves, through the introduction of a shape parameter *p* (Equation (9)):

(9) $$\log\;(N)\;=\;\log\left(N_{0}\right)\;-{\left(\frac{t}{\delta }\right)}^{p}$$

Here, *δ* denotes the time required for the first log reduction in microbial numbers, and *p* reflects the curve’s shape: *p* < 1 indicates a concave curve (tailing), while *p* > 1 suggests a convex curve (shouldering) [24].

Moreover, in predictive microbiology, the kinetic parameters of these primary inactivation models are frequently linked to environmental factors—such as temperature, pH, or chemical concentration—using secondary models. A common form of secondary modelling for inhibition data expresses parameters like *k* or *δ* as logarithmic function of an independent environmental variable (Equation (10)):

(10) $$k\;or\;\delta =a-b*log\left(Independent\;variable\right)$$

where *a* and *b* are regression coefficients capturing the strength and direction of the environmental influence.

These models, alone or combined with secondary relations, enable detailed predictions of microbial survival under diverse processing conditions and are central tools for food safety, preservation strategy design, and risk assessment.

### 2.2. Machine Learning Models

The predictive power of a machine-learning model depends on how well it balances bias and variance in a given dataset. Support Vector Regression (SVR), a kernel-based, non-parametric extension of Support Vector Machines, maps the data into a high-dimensional space where linear or nonlinear relationships can be captured [14]. In this study we used the radial basis-function (RBF) kernel, which performs well in high-dimensional settings but can be sensitive to noisy data.

Decision Tree Regression (DTR) is an interpretable, tree-based algorithm that recursively splits the data on individual features [14]. It is fast, requires little preprocessing, and can handle categorical variables without encoding. Nevertheless, single trees often suffer from high bias and high variance; ensemble techniques are therefore commonly adopted to boost accuracy.

Gaussian Process Regression (GPR) offers a fully Bayesian, non-parametric alternative [25]. By treating the target function as a multivariate Gaussian distribution, GPR provides both mean predictions and credible intervals. Using a squared-exponential kernel, it captures smooth, continuous trends and delivers reliable estimates in sparsely sampled regions, although the need to invert a full covariance matrix makes it computationally expensive for large datasets.

Model performance was evaluated with 5-fold cross-validation, a compromise between unbiased error estimation and computational cost [26]. The data were partitioned into five folds; in each iteration four folds were used for training and one for testing, and the results were average. A simple hold-out split was avoided because it can give overly optimistic or pessimistic errors depending on how the data are divided [27].

All input features were standardized using the z-score method to ensure equal contribution to the models and to minimize bias from differences in scale. For datasets containing fewer than 750 samples, a resampling strategy was applied in which the original data were randomly resampled and uniform noise (±0.2 log CFU) was added to the target variable within the training folds. This procedure increased the effective sample size and reduced overfitting; however, it is acknowledged that label noise is equivalent to synthesizing new values and may alter the true error structure, potentially exaggerating robustness and interval coverage. This limitation was explicitly noted, while it was emphasized that with sufficiently large datasets the need for augmentation disappears and greater accuracy and robustness can be achieved. The pre-processed data were then divided into training and test sets using an 80/20 split. Three regression algorithms—Gaussian Process Regression (GPR), Support Vector Regression (SVR), and Random Forest Regression (RFR)—were each trained on identically pre-processed data. Through this comprehensive workflow, robust performance evaluation and predictive uncertainty quantification across all regression models were achieved.

### 2.3. Parameter Estimation and Uncertainty Quantification

Accurate modelling of microbial dynamics requires not only point estimates of kinetic parameters but also a clear statement of their uncertainty. Accordingly, we report standard errors and 95% prediction (or credible) intervals for every classical and machine-learning model on the platform.

For nonlinear least-squares fits, the parameter covariance matrix ***C*** was obtained as the inverse Hessian of the Levenberg–Marquardt algorithm [28,29]. The standard error of each parameter was calculated as Equation (11):

(11) $$\mathrm{SE}(\hat{\theta_{i}})\;=\;\sqrt{C_{ii}}$$

where C***_ii_*** is the i-th diagonal element of the covariance matrix C.

To propagate parameter uncertainty into prediction intervals, the gradient (Jacobian) of the fitted model with respect to its parameters was computed at each time point. The variance of the fitted curve $\hat{y}\left(t\right)$ was then estimated as:

(12) $$\sigma^{2\hat{ᵧ}\left(t\right)}={\left[\nabla f\left(t,\hat{\theta }\right)\right]}^{T}\times C\times \nabla f\left(t,\hat{\theta }\right)+s{ₑ}^{2}$$

where ∇*f* denotes the gradient of the model function with respect to the parameter vector *θ*, and *S_e_*^2^ is the residual variance, calculated as Equation (13):

(13) $$s_{e}^{2}=\;\frac{1}{n-p}\sum_{i=1}^{n}{\left(yi-y\hat{i}\right)}^{2}$$

with *n* being the number of data points and *p* the number of fitted parameters.

Pointwise 95% prediction intervals were constructed using: $\hat{y}\left(t\right)\pm 1.96\times \sigma_{\hat{y}}\left(t\right)$. In Gaussian Process Regression (GPR), the predictive variance is provided directly by the probabilistic model and was used [30] to define the 95% confidence bands as: $\hat{y}\left(t\right)\pm 1.96\times \sigma_{\mathrm{GPR}}\left(t\right)$.

For non-probabilistic models such as SVR and Random Forest Regression, prediction intervals were formally assessed using a non-parametric bootstrap approach with 100 resampling iterations. In each bootstrap replicate, the model was retrained on a randomly resampled training set, and predictions were generated for the test data. The bootstrap size was set to 100 to balance coverage with computational feasibility, and this choice may limit representation of the distribution tails. The lower and upper bounds of the 95% prediction interval were determined as the 2.5th and 97.5th percentiles of the aggregated bootstrap predictions, following the procedure outlined by Efron and Tibshirani [31]. This formal bootstrap-based uncertainty quantification was implemented for all relevant test cases and is reported alongside point predictions throughout the results. These intervals provide a robust, data-driven measure of predictive uncertainty, allowing users to better assess the reliability and practical significance of the model outputs.

This unified framework provides statistically rigorous confidence bounds for every fitted curve and parameter, thereby enhancing the transparency and reliability of predictive-microbiology outputs [32]. All models share a common unit system: microbial concentrations are reported as log CFU/g, the maximum specific growth rate (*µₘₐₓ*) in 1/h, and both time and lag-phase duration in hours.

### 2.4. Comparison of the Goodness of Fit

The comparison of the performance of the models was carried out by using the root mean square error (*RMSE*) and coefficient of determination (*R^2^*) using Equation (14) and Equation (15), respectively:

(14) $$RMSE=\sqrt{\sum_{i=1}^{n}\frac{{\left(x_{obs}-x_{fit}\right)}^{2}}{n-s}}$$

(15) $$R^{2}=1-\left(\frac{SSE}{SST}\right)$$

where *x_obs_* is the experimental bacterial growth, *x_fit_* is the fitted value, *n* is the number of experiments, *s* is the number of parameters of the model, *SSE* is the sum of squares of errors and *SST* is the total sum of squares.

### 2.5. Statistical Analysis

To evaluate whether there were statistically significant differences between the results obtained from the machine learning modelling approach and the traditional mechanistic modelling approach, the Wilcoxon signed-rank test was applied. This non-parametric test was selected because it does not assume a normal distribution of the data and is well suited for comparing paired observations, such as model performance metrics obtained from the same datasets under two different modelling strategies [33]. The analysis was performed using the signrank function in the statistical toolbox of MATLAB, version 8.3.0.532 (R2014a). A significance threshold of *p* ≤ 0.05 was adopted, meaning that differences between the two approaches were considered statistically significant when the probability of the observed differences occurring by chance was 5% or less.

### 2.6. Modelling Dataset and Preprocessing

The modelling dataset was compiled from microbial growth and inhibition studies reported in three peer-reviewed publications, each representing distinct food matrices and environmental conditions. For growth modelling, two independent datasets were used. The first, from Gospavic et al. [17], provided 44 individual data points for *Pseudomonas* spp. growth in fresh chicken breast fillets stored aerobically at isothermal conditions of 2, 4, 10, 15, and 20 °C. The fillets were prepared from freshly slaughtered and air-chilled chickens, portioned into 100 g samples, packaged in low-density polyethylene films, and incubated in precision temperature chambers, with microbial counts taken at regular intervals to produce growth curves. The second dataset, from Lytou et al. [18], contributed 33 growth data points describing the natural aerobic microbiota of unmarinated chicken breast fillets stored at 4, 10, and 15 °C, with daily total viable count (TVC) measurements used to capture uninfluenced microbial growth. For inhibition modelling, 36 data points were obtained from Possas et al. [19], who investigated the inactivation of *Listeria monocytogenes* on stainless steel food-contact surfaces using slightly acidic electrolyzed water (SAEW, pH 5.0) at chlorine concentrations of 50, 100, 150, and 200 mg/L over exposure times from 0 to 360 s. In these experiments, stainless steel coupons were inoculated with a four-strain cocktail of *L. monocytogenes*, allowed to attach, treated with SAEW, and survivors were enumerated. In total, the dataset comprised 113 microbial observations spanning growth and inactivation scenarios across two food matrices (chicken fillets and stainless-steel surfaces). For traditional modelling approaches (both one-step and two-step models), the raw data were used without any standardization. For machine learning modelling, all numerical features were standardized using z-score normalization calculated from the training set to ensure equal scaling and prevent bias from differing variable magnitudes. The dataset was split into training and test sets using an 80/20 ratio, with stratification applied to preserve the distribution of temperature and chlorine conditions across sets [26]. Model validation was performed using 5-fold cross-validation on the training data to balance computational efficiency with reliable error estimation. Regarding the composition of feature variables, traditional models used a single independent variable—temperature for growth modelling and chlorine concentration for inhibition modelling—reflecting standard kinetic modelling conventions. In contrast, machine learning models may incorporate multiple individual features to improve predictive performance and generalizability. For growth prediction, the input features included temperature, pH, water activity (a_w_), time, and, where applicable, the presence of antimicrobial agents. For inhibition modelling, the variables consisted of chlorine concentration, exposure time, pH, and surface type. This multi-feature input enabled machine learning algorithms to capture more complex and nonlinear relationships relevant to microbial behaviour under diverse food safety conditions.

## 3. Results and Discussion

This study developed a dynamic software platform integrating classical predictive microbiology models and machine learning (ML) approaches to model microbial growth and inhibition under various environmental conditions. To validate the platform’s accuracy and reliability, its performance was compared against published experimental data and literature models using multiple modelling strategies: two-step, one-step, and machine learning-based predictions.

As a first step in this evaluation, the two-step modelling approach implemented in the platform was assessed by comparing its predictions to experimental data reported by Gospavic et al. [17] and Lytou et al. [18], covering a range of temperatures. The corresponding results are summarized for Baranyi model in Table 1 and Table 2. In Table 1, comparing the modelling platform’s results with Gospavic et al. [17], the estimated kinetic parameters (*y*_0_, *y_max_*, and *μ_max_*) are generally in good agreement with the literature values across the tested temperatures (2 °C to 20 °C). For instance, at 20 °C, the platform estimated *μ_max_* as 0.243 ± 0.028 1/h, which is close to the literature value of 0.255 ± 0.028 1/h. Additionally, the RMSE values were consistently low, with the highest observed RMSE being 0.333 log CFU/g, indicating accurate curve fitting. R^2^ values remained high (0.974–0.991), demonstrating strong explanatory power of the fitted models.

Similarly, Table 2 shows the results for Lytou et al. [18], where the platform also closely replicated the experimental growth parameters. Although minor differences were observed—particularly in initial population (*y*_0_) estimates—the RMSE values from the modelling platform were notably lower than those from the literature. For example, at 4 °C, the platform achieved an RMSE of 0.090 log CFU/g, significantly lower than the literature value of 0.320 log CFU/g, reflecting improved precision in capturing growth dynamics. Furthermore, R^2^ values exceeded 0.95 in all cases, confirming the platform’s robustness.

These results indicate that the two-step modelling capability of the platform can successfully replicate published experimental observations, with comparable or improved fitting accuracy. The lower RMSEs suggest reduced residual variability, which may be attributed to modern optimization algorithms and robust error analysis implemented in the platform. Additionally, this modelling platform does not provide results for only a single primary model but also offers predictions using four different primary models—modified Gompertz, Logistic, Baranyi, and Huang—which are among the most commonly used models in predictive food microbiology. This feature allows users to directly compare the prediction capabilities of each primary model on the same dataset, supporting more informed model selection and enhancing the robustness of microbial growth predictions.

The performance of the one-step modelling approach was evaluated by comparing it with previous studies [13] as summarized in Table 3. Four primary growth models—modified Gompertz, Logistic, Baranyi, and Huang—were fitted simultaneously across varying temperatures to jointly estimate kinetic and environmental parameters. In all primary models, the platform’s estimated parameters were generally consistent with literature values. For instance, in the modified Gompertz model, the platform estimated *y*_0_ as 3.97 ± 0.34 log CFU/g and *T_min_* as −9.24 ± 0.84 °C, closely matching the literature values (*y*_0_ = 3.97 ± 0.34, *T_min_* = −9.10 ± 0.80 °C). Similarly, estimates of *b*_1_ and *b*_2_ coefficients showed good alignment, with minor deviations, especially in the Huang model, where the platform estimated *b*_2_ as 2.41 ± 0.58, slightly higher than the literature’s 0.80 ± 0.70. However, these differences were within acceptable confidence intervals, suggesting robustness of the platform’s one-step fitting process. The RMSE values obtained by the platform ranged between 0.530 and 0.551 log CFU/g across all models, generally lower than those reported in the literature (0.548 to 0.570), indicating improved model fitting accuracy. R^2^ values remained high (>0.924), confirming that the platform effectively captured growth kinetics under dynamic conditions.

One-step modelling for growth behaviour offers significant advantages over the traditional two-step approach, particularly in reducing error propagation between primary and secondary models and ensuring more stable parameter estimation. The platform’s successful implementation of one-step modelling highlights its capacity to handle complex datasets and provides users with an alternative method for more integrative and biologically consistent modelling.

The two-step modelling of microbial inhibition was validated against the study of Possas et al. [19], focusing on the inactivation of microorganisms under varying concentrations of available chlorine. Results are summarized in Table 4.

At all tested chlorine concentrations (50–200 mg/L), the platform’s predictions for δ (time for first log reduction) and the shape parameter *p* were closely aligned with the literature data. For example, at 50 mg/L, the platform estimated *δ* as 20.43 ± 9.70 s and *p* as 0.33 ± 0.05, consistent with the literature values of 19.91 ± 4.50 s and 0.33 ± 0.03, respectively. RMSE values for the platform ranged between 0.113 and 0.294 log CFU/g, generally lower than or comparable to literature values, indicating accurate curve fitting. R^2^ values remained high (>0.92), suggesting excellent model performance. These results underscore the platform’s ability to reliably replicate microbial inactivation kinetics using the two-step approach, even under conditions of chemical stressors. The improved or comparable RMSE values reflect the efficiency of the implemented numerical methods and robust error analysis. Table 5 presents the performance of the platform’s one-step inhibition modelling compared to the study by Possas et al. [19]. The one-step model jointly estimated kinetic parameters and environmental effects in a single optimization step.

The platform’s predictions were highly consistent with the literature, with parameter estimates falling within overlapping confidence intervals. For instance, *δ* was estimated as 0.33 ± 0.03 s, closely matching the literature value of 0.33 ± 0.02 s. Similarly, the regression coefficients a and b were estimated as 64.49 ± 25.02 and 26.92 ± 10.41, respectively, which are comparable to literature values (60.26 ± 11.73 and 25.16 ± 4.86). The RMSE achieved by the platform was slightly lower (0.274 vs. 0.286 log CFU/g), reflecting slightly improved fitting accuracy. These findings demonstrate that the one-step approach effectively captures the dynamic interplay between microbial inactivation kinetics and environmental variables, reducing fitting errors and enhancing biological interpretability. The consistency of parameter estimates further validates the reliability of the platform.

To assess the potential of data-driven approaches, the predictive capabilities of three machine learning models—Gaussian Process Regression (GPR), Support Vector Regression (SVR), and Random Forest Regression (RFR)—were compared for modelling microbial growth and inhibition, as summarized in Table 6. The results demonstrate that machine learning methods can effectively capture the complex, nonlinear relationships inherent in microbiological data, offering a valuable alternative or complement to traditional mechanistic models.

For microbial growth prediction, GPR delivered outstanding performance, achieving low RMSEs of 0.251 on the training set and 0.326 on the test set, along with high R^2^ values of 0.986 and 0.974, respectively. This indicates excellent predictive accuracy and minimal overfitting, highlighting GPR’s strength in handling nonlinear patterns and providing reliable estimates even in regions with sparse data. RFR performed nearly as well, with RMSEs and R^2^ values closely matching those of GPR, suggesting that ensemble tree-based methods are equally effective for modelling microbial growth dynamics due to their capacity to capture complex interactions among environmental variables. In contrast, SVR, while still showing satisfactory performance, exhibited higher RMSEs (0.668 in training and 0.652 in testing) and lower R^2^ values around 0.90 (Table 6). This indicates slightly reduced predictive accuracy, which may stem from SVR’s sensitivity to noise and its reliance on kernel parameters that require careful tuning. Nonetheless, SVR still offers valuable insights and could be beneficial in scenarios where model interpretability and computational efficiency are priorities.

For microbial inhibition modelling, both Gaussian Process Regression (GPR) and Random Forest Regression (RFR) delivered highly accurate predictions, achieving low RMSEs of approximately 0.105 on the training data and around 0.119 on the test data, with R^2^ values consistently exceeding 0.99. These results confirm their excellent capability in modelling microbial inactivation kinetics and capturing the complex, nonlinear dynamics inherent in inhibition data. In contrast, Support Vector Regression (SVR) displayed lower predictive performance, with higher RMSEs and R^2^ values around 0.91, suggesting that while SVR can provide reasonable estimates, it may be less robust for modelling inhibition behaviour, particularly when dealing with complex nonlinear interactions among multiple environmental factors (Table 6).

Overall, ML models, especially GPR and RFR, outperformed traditional parametric approaches in terms of flexibility and predictive accuracy, particularly for complex, multidimensional datasets. ML methods inherently capture nonlinearities and interactions without the need for predefined mathematical formulations, making them highly adaptable for predictive microbiology applications. However, their computational demands, interpretability challenges, and potential overfitting risks should be carefully considered.

The comparative results indicate that the developed platform achieves robust modeling performance across both classical (two-step and one-step) and machine learning approaches. For multiple datasets, the platform consistently produced lower RMSE values and higher R^2^ scores—often exceeding 0.90—compared to values reported in the literature. These performance metrics demonstrate the platform’s effectiveness in accurately replicating, and in some cases improving upon, published results. Notably, classical models provide strong mechanistic insights and remain highly interpretable, making them invaluable for understanding underlying microbial kinetics and for use in regulatory or risk assessment contexts where transparency is essential. However, machine learning (ML) models, including SVR, RFR, and GPR, were found to offer superior predictive power, particularly in scenarios involving complex, nonlinear interactions among environmental factors. This was further confirmed by the Wilcoxon signed-rank test (*p*-value < 0.05), which revealed a statistically significant improvement in prediction performance for ML-based models over traditional modelling approaches for both growth and inhibition datasets. While this trade-off highlights the increased accuracy of ML models, it also underscores the importance of selecting the appropriate modelling strategy based on the intended application and the need for interpretability versus prediction accuracy.

Although several tools exist for microbial model fitting, their scope remains fragmented. While ComBase (via DMFIT) is often regarded as the reference resource for predictive microbiology, its modelling capacity is narrowly confined. DMFIT can only fit a single primary growth model (Baranyi) and lacks secondary modelling capabilities, which limits its applicability when environmental drivers must be considered. Growth Predictor extends functionality by enabling secondary modelling, but only through the Baranyi equation as the primary model and via the conventional two-step approach, where error propagation remains a critical weakness [34]. CardinalFit, conversely, focuses exclusively on secondary models and cannot handle primary model fitting [35]. GInaFiT addresses only microbial inactivation processes under static conditions [36]. Collectively, these platforms remain fragmented—none allow side-by-side comparison of multiple primary models for growth and inactivation, none offer evaluation of both one-step and two-step approaches, and none incorporate machine learning to capture nonlinear, multidimensional interactions.

By contrast, the modelling platform developed in this study supports comparative fitting across diverse primary models for both growth and inactivation, integrates both one-step and two-step approaches, and incorporates machine learning methods that transcend the structural constraints of classical models. This integration not only addresses the limitations of ComBase/DMFIT and other existing tools but also provides a genuinely novel framework that bridges traditional predictive microbiology with modern, data-driven methodologies. Table 7 summarizes the functions of existing tools in comparison with the present platform, distinguishing between currently implemented features and those planned for future development.

Some limitations should also be acknowledged. For example, the current machine learning models in the platform are restricted to two features (time plus one independent variable). Future versions will be designed to incorporate more complex architectures that can handle a higher number of simultaneous features. Moreover, the performance of the platform remains dependent on the size and representativeness of the available datasets, and its predictive accuracy may be affected by noise, missing values, and inconsistencies in data formats. Although initial outlier handling and simple data augmentation methods are already implemented, further improvements—such as advanced imputation techniques and more robust regression approaches—are needed to enhance reliability in real-world applications. Future work should focus on incorporating larger and more diverse public databases (e.g., ComBase), developing interpretable machine learning or hybrid models that combine mechanistic and data-driven approaches, and implementing automated hyperparameter optimization. These advancements will help balance the strengths of classical and ML models, ultimately improving the platform’s utility for researchers, industry professionals, and regulatory authorities in predicting and controlling microbial dynamics under diverse and challenging conditions.

## 4. Conclusions

This study presents a dynamic modelling platform that integrates classical predictive microbiology with modern machine learning in a single, interactive environment. By combining multiple primary growth and inactivation models with machine learning regressors, it enables side-by-side comparisons of modelling approaches and provides flexibility for different microbial behaviours and conditions. The study is limited by the relatively small dataset, and the findings should therefore be viewed as illustrative rather than universal. Even within this scope, the platform highlights the potential of uniting mechanistic interpretability with data-driven flexibility, supported by tools such as uncertainty quantification, visual diagnostics, and reproducible workflows. Broader validation with larger and more diverse datasets will be needed to confirm generalizability. Future work will focus on incorporating public databases, developing hybrid models, and improving robustness, thereby advancing the platform’s utility for food safety modelling and risk assessment.

## Figures and Tables

**Table 1:** Comparison of growth modelling predictions of the developed platform using the two-step modelling approach for the data reported by Gospavic et al. [17].

Source	Temperature (°C)	*y*_0_ (log CFU/g)	*y_max_* (log CFU/g)	*µ_max_* (1/h)	RMSE	R^2^
Gospavic et al. [17]	2	3.97 ± 0.32	10.15 ± 0.46	0.026 ± 0.005	0.371	0.982
	4	3.95 ± 0.30	9.73 ± 0.35	0.043 ± 0.008	0.338	0.986
	10	3.53 ± 0.33	10.14 ± 0.78	0.081 ± 0.015	0.395	0.980
	15	4.45 ± 0.26	9.51 ± 0.23	0.236 ± 0.042	0.329	0.986
	20	3.22 ± 0.15	8.40 ± 0.18	0.255 ± 0.028	0.153	0.996
Modelling platform	2	3.71 ± 0.85	9.94 ± 0.29	0.024 ± 0.003	0.274	0.984
	4	3.83 ± 0.70	9.61 ± 0.32	0.035 ± 0.005	0.285	0.982
	10	3.32 ± 2.33	9.87 ± 0.57	0.076 ± 0.010	0.333	0.974
	15	4.24 ± 0.58	9.46 ± 0.21	0.178 ± 0.022	0.220	0.987
	20	3.03 ± 0.94	8.19 ± 0.17	0.243 ± 0.028	0.168	0.991

**Table 2:** Comparison of growth modelling predictions of the developed platform using the two-step modelling approach for the data reported by Lytou et al. [18].

Source	Temperature (°C)	*y*_0_ (log CFU/g)	*y_max_* (log CFU/g)	*µ_max_* (1/h)	RMSE	R^2^
Lytou et al. [18]	4	5.12 ± 0.20	9.84 ± 0.12	0.088 ± 0.011	0.320	0.967
	10	5.26 ± 0.12	9.86 ± 0.16	0.155 ± 0.021	0.363	0.955
	15	5.67 ± 0.22	9.79 ± 0.17	0.233 ± 0.033	0.368	0.935
Modelling platform	4	4.51 ± 0.29	9.59 ± 0.05	0.045 ± 0.002	0.090	0.997
	10	4.78 ± 0.79	9.77 ± 0.11	0.062 ± 0.010	0.234	0.979
	15	4.75 ± 1.19	9.35 ± 0.13	0.085 ± 0.026	0.296	0.955

**Table 3:** Comparison of growth modelling predictions of the developed platform using the one-step modelling approach for the pooled studies of Gospavic et al. [17] and Lytou et al. [18].

Source	Primary Model	*y*_0_ (log CFU/g)	*y_max_* (log CFU/g)	*T_min_* (°C)	*b*_1_ (1/h.°C^2^)	*b*_2_ (-)	RMSE	R^2^
Tarlak and Pérez-Rodríguez [13]	Modified Gompertz	3.97 ± 0.34	9.66 ± 0.13	−9.10 ± 0.80	0.0014 ± 0.0002	1.30 ± 1.20	0.548	0.930
	Logistic	3.42 ± 0.16	9.60 ± 0.11	−9.10 ± 0.80	0.0014 ± 0.0001	0.00 ± 0.00	0.553	0.928
	Baranyi	4.13 ± 0.24	9.52 ± 0.11	−9.10 ± 0.80	0.0011 ± 0.0001	0.60 ± 0.90	0.570	0.924
	Huang	4.24 ± 0.13	9.52 ± 0.11	−9.10 ± 0.80	0.0011 ± 0.0001	0.80 ± 0.70	0.563	0.926
Modelling platform	Modified	3.97 ± 0.34	9.66 ± 0.13	−9.24 ± 0.84	0.0014 ± 0.0002	1.29 ± 1.20	0.530	0.933
	Logistic	3.42 ± 0.54	9.60 ± 0.13	−9.10 ± 0.81	0.0014 ± 0.0002	0.00 ± 1.79	0.535	0.932
	Baranyi	3.85 ± 0.60	9.52 ± 0.11	−9.06 ± 0.77	0.0011 ± 0.0001	0.00 ± 1.81	0.551	0.928
	Huang	4.29 ± 0.14	9.56 ± 0.11	−7.25 ± 0.85	0.0017 ± 0.0002	2.41 ± 0.58	0.533	0.933

**Table 4:** Comparison of prediction inhibition behaviour of modelling platform using the two-step modelling approach for the data reported by Possas et al. [19].

Source	Available Chlorine Concentration (mg/L)	*δ* (s)	*p* (-)	RMSE	R^2^
Possas et al. [19]	50	19.91 ± 4.50	0.33 ± 0.03	0.130	0.980
	100	4.35 ± 2.93	0.27 ± 0.05	0.288	0.920
	150	4.32 ± 2.36	0.30 ± 0.04	0.284	0.930
	200	3.91 ± 1.66	0.37 ± 0.04	0.334	0.960
Modelling platform	50	20.43 ± 9.70	0.33 ± 0.05	0.113	0.980
	100	4.12 ± 4.93	0.27 ± 0.07	0.254	0.927
	150	5.02 ± 4.82	0.31 ± 0.06	0.254	0.942
	200	3.59 ± 2.87	0.37 ± 0.06	0.294	0.967

**Table 5:** Comparison of prediction inhibition behaviour of modelling platform using the one-step modelling approach for the data reported by Possas et al. [19].

Source	*a* (s)	*b* (s)	*δ* (s)	RMSE	R^2^
Possas et al. [19]	60.26 ± 11.73	25.16 ± 4.86	0.33 ± 0.02	0.286	0.950
Modelling platform	64.49 ± 25.02	26.92 ± 10.41	0.33 ± 0.03	0.274	0.954

**Table 6:** Comparison of the predictive capabilities of machine learning models for microbial growth and inhibition.

Microorganism Behaviour	Machine Learning Model ^c^	Set	RMSE	R^2^
Growth ^a^	GPR	Train	0.251	0.986
		Test	0.326	0.974
	SVR	Train	0.668	0.904
		Test	0.652	0.897
	RFR	Train	0.252	0.986
		Test	0.328	0.974
Inhibition ^b^	GPR	Train	0.105	0.993
		Test	0.119	0.991
	SVR	Train	0.385	0.912
		Test	0.368	0.912
	RFR	Train	0.105	0.993
		Test	0.119	0.991

^a^ Growth data obtained from the pooled studies of Gospavic et al. [17] and Lytou et al. [18]. ^b^ Inhibition data obtained from the Possas et al. [19]. ^c^ Gaussian Process Regression (GPR), Support Vector Regression (SVR), and Random Forest Regression (RFR).

**Table 7:** Comparison of existing microbial modelling tools and functions of the present platform (implemented vs. planned).

Tool	Scope	Limitations
ComBase/DMFIT	Fits Baranyi growth model (primary only)	No secondary modelling; limited to a single model
Growth Predictor	Baranyi model with secondary modelling (two-step only)	Error propagation; limited flexibility
CardinalFit	Fits secondary models (cardinal parameter approach)	Cannot handle primary model fitting
GInaFiT	Fits inactivation models (e.g., log-linear, Weibull) under static conditions	Growth modelling not supported
**Our Platform**	**Implemented Functions**	**Planned Functions**
Comparative model fitting	Multiple primary models (growth & inactivation), one-step and two-step approaches	Integration with additional public microbial databases (e.g., ComBase)
Machine learning	SVR, RFR, GPR models for both growth and inactivation, with uncertainty quantification	Development of hybrid mechanistic–ML models for improved interpretability
Usability	Unified web interface and API, interactive diagnostics, exportable reports, reproducible workflows	Automated hyperparameter optimization, expanded visualization options

## Data Availability

The original contributions presented in this study are included in the article. Further inquiries can be directed to the corresponding author.
